# Let’s Play Cards: Multi-Component Cognitive Training With Social Engagement Enhances Executive Control in Older Adults

**DOI:** 10.3389/fpsyg.2018.02482

**Published:** 2018-12-06

**Authors:** Chun-Yu Kuo, Yang-Ming Huang, Yei-Yu Yeh

**Affiliations:** ^1^Department of Adult & Continuing Education, National Taiwan Normal University, Taipei, Taiwan; ^2^Department of Psychology, National Taiwan University, Taipei, Taiwan; ^3^Department of Psychology, Fu Jen Catholic University, New Taipei City, Taiwan

**Keywords:** cognitive training, healthy older adults, executive function, cognitive control, social engagement

## Abstract

Cognitive training and social engagement are two of the routes that potentially improve cognitive functions in older adults. The former targets specific functions so that an intervention can trigger the plasticity and efficiency of the underpinning neural systems, and the latter also provides an environment supportive of social and emotional needs. We investigated whether an integration of the two routes could enhance cognitive functions related to executive control, because no prior research has adopted a theory-driven approach to design a group-based cognitive training program for executive control. Forty-six healthy and active older adults living in community settings were randomly assigned to a group-based training program or a group-based active control program. Twenty-three volunteers in a community center were recruited for the waitlist control group. A battery of card games was designed for the cognitive training program based on three theoretical models of executive functions. A set of commercial board games were run in the active control program. Using untrained tests as the outcome measures, we found significant improvement on executive control in the cognitive training group compared with the active and waitlist control groups while the two control groups did not differ in performance. The cognitive training group did not outperform the two groups on a test of reasoning or on a test of delayed episodic memory. The results support the idea that cognitive training with social interaction can improve performance on untrained tests that share overlapping cognitive processes. Despite the inability to adapt to each person’s performance, integrating the two routes is beneficial for improving cognitive functions in older adults.

## Introduction

Maintaining physical and mental health in an aging population is increasingly important for societies worldwide. Cognitive decline that can compromise mental health and the ability to live independently drives efforts to seek interventions that preserve or enhance cognitive abilities. While a plethora of empirical research has been devoted to interventions that preserve or enhance brain health (see Bamidis et al., 2014; Ballesteros et al., 2015 for reviews), few studies have integrated multi-component cognitive training with social engagement as a choice for an aging, heterogeneous population. Here, we designed a battery of card games targeting cognitive functions related to executive control and investigated whether this integrative cognitive training could improve executive control compared with both an active-control group and a waitlist-control group.

Population aging imposes great challenges for societies worldwide in terms of health care, infrastructure, and social costs. In addition to physical health, a key to successful aging is to maintain brain health (Reuter-Lorenz and Park, 2014). To achieve this aim, interventions, via a variety of physical and mental activities, can be incorporated in the everyday life of the aging population. Exercise, new learning, engagement, and cognitive training are four proposed channels to maintain brain functions potentially by enhancing neural activity (Reuter-Lorenz and Park, 2014). This contention is supported by studies that show the neural effects of exercise training (Erickson et al., 2011; Jonasson et al., 2017), dance (Burzynska et al., 2017), new learning (McDonough et al., 2015), social engagement (Carlson et al., 2009, 2015), and cognitive training (Strenziok et al., 2014; Kim, 2015; Beauchamp et al., 2016; Cao et al., 2016; Jiang et al., 2016; Thompson et al., 2016; Heinzel et al., 2017; Küper et al., 2017; Motes et al., 2018).

## Multi-Domain Intervention

Given that individual interventions can be effective for enhancing neural activity, it is not surprising that multi-domain interventions have become a new and promising approach for maintaining brain functions (see Bamidis et al., 2014; Ballesteros et al., 2015 for reviews). Multi-domain interventions can be classified by whether the intervention adds a new element to an existing intervention. Without adding a new element, Tai chi and dance are two interventions that consist of a moderately intensive level of physical activity while requiring mental effort to remember the postures and sequences with concentration. Empirical studies have shown the benefits of Tai chi (see Miller and Taylor-Piliae, 2014; Zheng et al., 2015 for reviews) and dance (Kattenstroth et al., 2013) for cognitive functions.

A second category of multi-domain intervention is to add a new element to an existing intervention. A combination of cognitive and physical interventions is popular, as it can promote both physical and mental fitness. A combined intervention has advantages over pure physical exercise (see Zhu et al., 2016 for a review). Exergaming, for example, integrates challenging cognitive activities in exercise training (Anderson-Hanley et al., 2012; Bamidis et al., 2015). After a long period of training, this combined intervention enhanced executive function (Anderson-Hanley et al., 2012) and global cognition, which includes measures of episodic memory, working memory, and executive function (Bamidis et al., 2015). Another example is a mix of different interventions in a quasi-educational program that combines new learning (handicraft making) with physical and health exercises and teaches the rules and strategies used in performing standardized cognitive tasks for solving daily problems (Cheng et al., 2012). This multi-domain intervention increases functional connectivity among brain regions (Cao et al., 2016) and modulates cortical thickness in related regions (Jiang et al., 2016) after 1 year of training.

## Social Engagement

Not all older adults can endure physical interventions at a moderate or an intensive level. A variety of multi-domain interventions is needed to fulfill the needs of a heterogeneous population. Social engagement with mental stimulation provides an alternative channel for older adults in community settings to maintain or enhance cognitive functions. Interventions in a socially interactive context may be ideal for maintaining cognitive functions while partially fulfilling emotional and social needs of community-dwelling older adults. Addressing emotional and social needs may be important for enhancing executive functions (Diamond and Ling, 2016), which are important for effective functioning in daily life (see Royall et al., 2007 for a review).

Correlational studies have shown that social ties and emotional connections with others influence healthy aging (see Ballesteros et al., 2015 for a review). Volunteer work with social interaction improved cognitive functions for those who showed impairment on attentional switching compared to a waitlist control group (Carlson et al., 2008). However, an empirical study showed that social engagement alone may not be beneficial for enhancing cognitive functions of healthy older adults (Park et al., 2014). Receptive social engagement without the involvement of active skill acquisition marginally improved processing speed compared with the control group. Productive social engagement, involving complex activities that demand the synergy of multiple cognitive functions, is necessary for improving cognitive functions (Chan et al., 2014; Park et al., 2014; Myhre et al., 2017; Vaportzis et al., 2017). Productive social engagement targeting divergent thinking also significantly improves this trained ability (Stine-Morrow et al., 2014).

## Cognitive Training with Social Engagement

Previous studies of cognitive training and social engagement have focused on complex skills in new learning (Chan et al., 2014; Park et al., 2014; Myhre et al., 2017; Vaportzis et al., 2017) or in solving ill-defined problems (Stine-Morrow et al., 2014). Few studies have investigated the possibility of targeting specific cognitive functions in a socially interactive context. One promising candidate for integrating multi-component cognitive training and social engagement is the usage of card games or board games in an intervention. The results of a correlational study revealed that board game players showed less decline in global functioning, measured by the Mini-Mental State Examination during the 20 years of follow-up, than non-board game players (Dartigues et al., 2013). Additionally, using games designed to train executive functions, an empirical study showed that the gaming group improved on executive control assessed by untrained tests of executive control in healthy older adults (Fissler et al., 2013). However, the details of the cognitive training program are not clearly described, a passive control group was used for comparison with the training group, and the sample size was small (9 in the training group and 8 in the control group). Therefore, whether multi-component cognitive training in a socially interactive context^1^ can enhance cognitive functions in older adults remains unexplored.

## The Current Study

The aim of this study is to investigate whether multi-component cognitive training in a socially interactive context can be effective for improving cognitive functions. The results should provide insights for designing interventions for community-dwelling older adults to satisfy cognitive and emotional needs. It has been suggested that multi-component cognitive training may provide more advantages than single-component cognitive training (Rebok et al., 2007; Eckroth-Bucher and Siberski, 2009; Gates and Valenzuela, 2010; Kueider et al., 2012; Gates and Sachdev, 2014; Binder et al., 2015). Supporting this view, a behavioral study showed that multi-component computerized training that integrates cognitive inhibition, visuomotor, and spatial navigation abilities provides greater improvement on the composite measure of attentional control than single-component interventions (Binder et al., 2016). Learning memory strategies and practicing tasks that focus on processing speed, selective attention, short-term memory span, verbal fluency, arithmetic, and reasoning also improve response accuracy and modulate neural activity that supports task-switching (Küper et al., 2017).

We designed a multi-component cognitive training program that primarily targets executive control, which consists of multiple cognitive functions. We define executive control as an umbrella term comprising attentional and control processes required to carry out goal-directed behavior. Cognitive control mechanisms support linguistic and non-linguistic performance in young adults (Hussey et al., 2017) and are identified as candidates for providing neural scaffolding that supports cognition across a broad range of tasks (Zokaei et al., 2017). Moreover, deficient modulation of the neural mechanisms of top-down control explains age-related decline in working memory (Heinzel et al., 2017), and a meta-analysis study showed that the neural mechanisms underlying executive control are recruited for compensatory scaffolding in older adults (see Li et al., 2015 for a review). According to the neural scaffolding framework (Reuter-Lorenz and Park, 2014; Zokaei et al., 2017), multi-component cognitive training that engages multiple functions of executive control is hypothesized to benefit performance on untrained tests that involve similar neural mechanisms. According to the cognitive reserve hypothesis (Whalley et al., 2004), this type of cognitive training could enhance the efficiency of cortical circuits sub-serving the involved cognitive functions via repeated use. We aimed to investigate whether this goal is achievable in a socially interactive context.

To integrate processes related to executive control in the games, we considered three theoretical models. In the multi-component model of working memory (Baddeley, 2012), the central executive module plays important roles in focusing attention/inhibition, switching, dividing attention, and interfacing with long-term memory (Baddeley, 1998). In a model of executive function (Miyake and Friedman, 2012), three mechanisms are included: a common mechanism that underlies inhibition, updating, and switching; an updating-specific mechanism; and a switching-specific mechanism. In Diamond’s (2013), three levels may characterize executive functions: working memory and inhibitory control at a basic level; cognitive flexibility in switching at an intermediate level; and reasoning, problem solving, and planning at a high level.

We took elements from each model in designing the formal cognitive training program: focusing attention, interfacing with long-term memory, dividing attention between two task sets, working memory, updating, reasoning, and problem solving. Each cognitive training game engages multiple-component cognitive functions underlying executive control^2^. Game difficulty was adapted according to the efficiency shown in most of the group members. An active control group went through training of eye-hand coordination and strategic thinking in a socially engaging atmosphere using commercial board games. Compared with the cognitive training games, the aspects related to the control of attentional deployment in these board games were exercised to a lesser degree. A waitlist control group included participants who actively volunteered in community services. Formal volunteering over time is associated with higher levels of cognitive function (Anderson et al., 2014; Guiney and Machado, 2017; Proulx et al., 2017 for reviews).

## Materials and Methods

### Participants

The research protocol was reviewed and approved by the Research Ethics Committee at the National Taiwan University. Volunteers were recruited from three community centers in Taipei, Taiwan. In two centers, news about the study was announced in the community newspaper and in a presentation to senior groups, and the center manager helped spread the news. Participants in both centers were informed that the aim of the study was to investigate how cognitive training activities could enhance cognitive functions.

Volunteers in these two centers who were willing to commit to an 8-week training program with two meetings per week were screened. Study participation exclusion criteria included the following: younger than 65 years old; substantial functional impairment (IADL disabilities) or cognitive decline (Mini-Mental State Examination [MMSE] score < 23); self-reported diagnosis of Alzheimer’s disease; medical conditions associated with imminent functional decline or death; severe loss in vision, hearing, or communicative ability that would interfere with study participation; and participation in other cognitive or physical training programs. Three participants were excluded because of low MMSE scores. Given the difficulty in recruiting participants, we recruited only 23 participants for each group, which fulfilled the goal of including a minimum of 20 subjects per study condition to have sufficient power to detect most effects (Simmons et al., 2011). This sample size is comparable to the one adopted in a recent study that investigated the effects of a multi-component cognitive training program relative to the effects of three single-component training interventions (Binder et al., 2016). A total of 69 participants provides a power of 0.92 to detect a medium-sized effect ($\eta_{p}^{2}$ = 0.15).

Forty-six participants in these two centers were randomly assigned to the training and active control groups. Participants were blind to the principles behind the design of the training exercises and were unaware of training in the other groups. Participants in both groups were told that the training exercises had the potential to protect them from cognitive decline. They received a NT$3000 (approximately US$100) monetary reward at the end of the posttest. Twenty-three participants in the waitlist control group were recruited from the volunteers in the third community center and the same criteria were used for exclusion. All screened participants met the inclusion criteria. They received a NT$500 (approximately US$16.67) monetary reward at the end of the posttest. All participants had normal or corrected-to-normal vision, and there is no significant difference among the three groups in age (*p* = 0.94), gender ratio (*p* = 0.28), level of education (*p* = 0.56), MMSE (*p* = 0.38), or IADL scores (*p* = 0.36) (Table 1).

**Table 1:** Demographic information for participants in all three conditions.

	*N*	Female participants (%)	Age (years)	Education level (years)	MMSE score	IADL score
Training group	23	65%	73.91 (6.07)	10.83 (4.38)	26.09 (1.68)	22.65 (1.27)
Active control group	23	70%	73.52 (6.21)	10.85 (3.54)	26.00 (1.73)	22.30 (1.94)
Waitlist control group	23	48%	73.30 (5.44)	9.70 (3.43)	26.65 (1.70)	21.91 (1.93)


### Trainers and Assessor

Four trainers were recruited based on colleagues’ recommendations of students who interact well with older adults. They had no background knowledge of cognitive training and were told that the study investigated how older adults interact in performing a variety of tasks. The only instruction was to engage and motivate all group members as much as possible in playing the games and resolve any conflicts if they arise. Trainers were randomly assigned to either the training group or the active control group without knowledge of which group was the experimental group. They were then trained on the activities involved in each game. At the end of each session, they reported the dynamics, general mood states, feedback received from the group members, and issues related to the adjustment of game difficulty. They received an hourly pay of NT$250 (approximately US$16.67) for their assistance.

A research assistant without background knowledge of introductory psychology administered the pretest and posttest. This assistant, who did not participate during the training period, knew that two forms of activities were trained. Expecting that the assistant could learn the group membership during rapport-building with the participants, we instructed the assistant to maintain neutrality and conduct the tests with consistent procedures across participants.

### Materials

**FIGURE 1:**
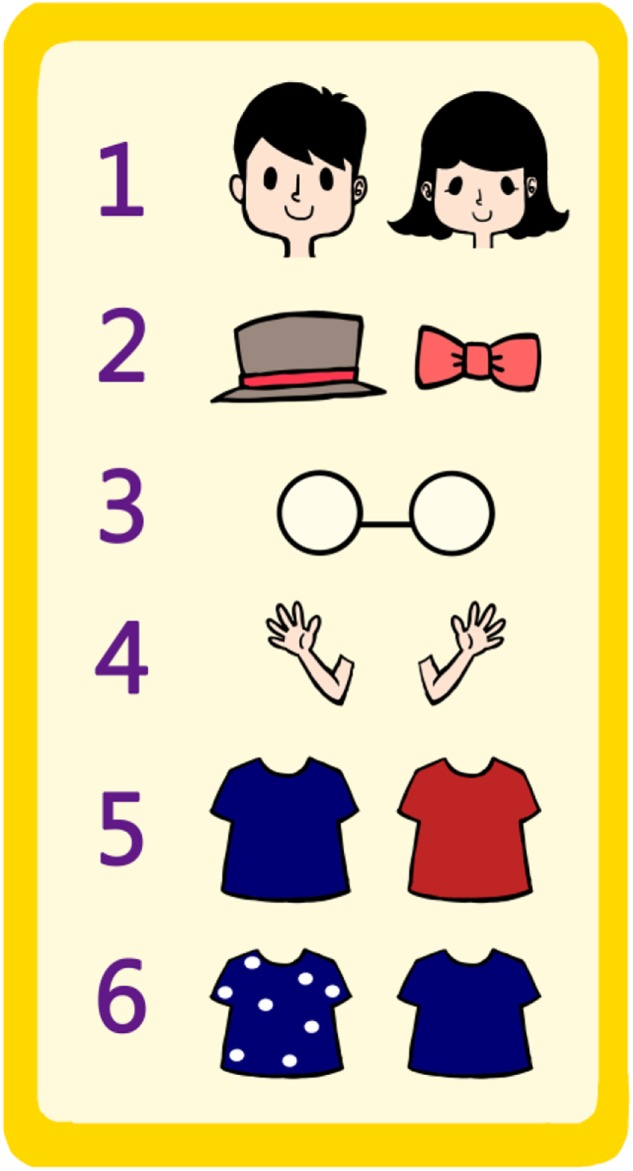
An illustration of the features shown on the cards. The head accessory varies according to the gender of the child, with a hat for a boy and a hairpin for a girl.

**FIGURE 2:**
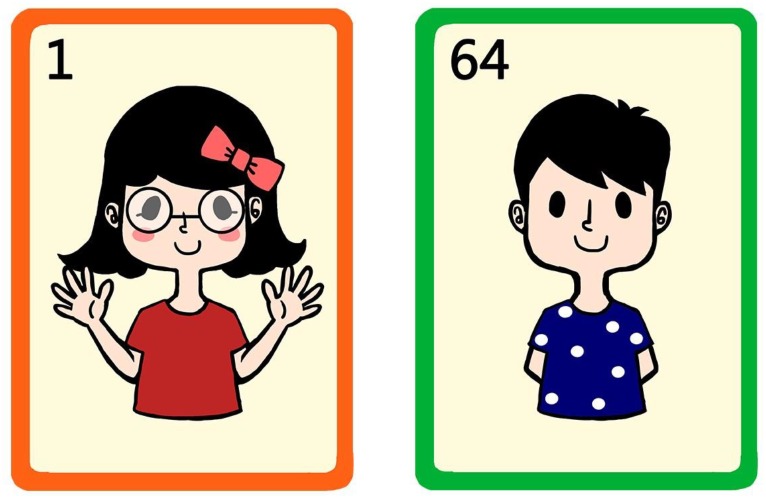
An example of two cards that differ on all six features.

**FIGURE 3:**
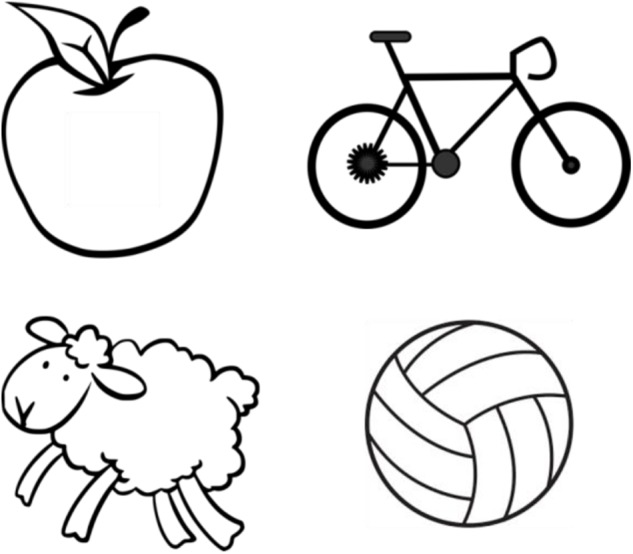
Examples of the cards used for the sorting activity that encourages interfacing with long-term memory.

**FIGURE 4:**
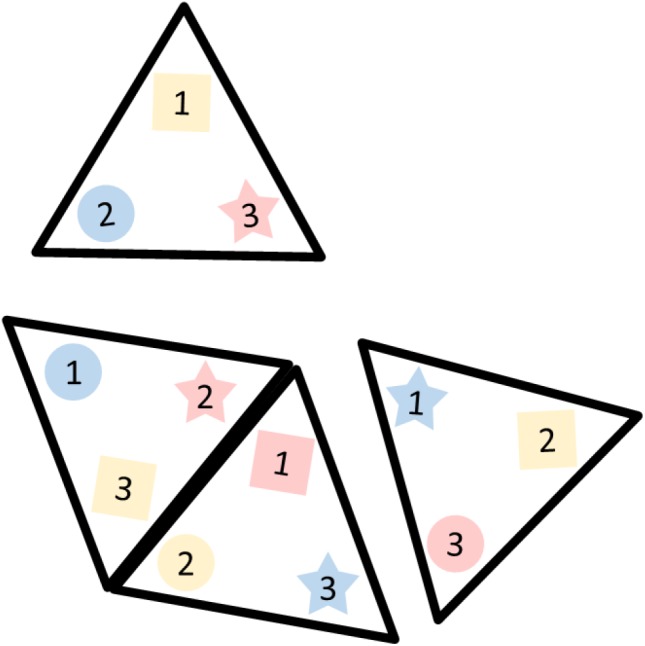
An illustration of the cards used for training the switching function in the warmup period.

Three decks of cards were designed for the training group. The first deck was used in most of the training activities. Each card has six features, including the gender of the child, head accessory, glasses, hands, color of t-shirt and pattern of the t-shirt (Figure 1). Each feature has two levels; therefore, the deck is composed of 64 unique cards. On the top left of each card is a unique number ranging from 1 to 64. Each card differs from every other card on at least one, and up to six, features (Figure 2). We chose these six dimensions with binary features for two reasons. These dimensions are frequently observed in everyday life so that the participants could relate their experiences. The binary features oppose each other so that selection of one feature on a dimension involves ruling out the other possibility. Multiple features on a dimension were not used in the material to keep the interfeature interference reasonable for older adults. The second deck was designed for demanding access to semantic knowledge stored in long-term memory with an image illustrated on each card (Figure 3). The third deck was used in warmup activities during two sessions. This deck consists of triangular cards with a colored shape (square, circle, or star) on the three corners and a digit (1, 2, or 3) inside the shape (Figure 4).

### Card Games for the Training Group

The cognitive training games consist of eight modules targeting selective attention via a visual search, interfacing with long-term memory via sorting, dividing attention between two task sets via dual-task activities, working memory via delayed-matching-to-sample, updating via an *n*-back game, reasoning via goal-setting, arithmetic and hypothesis testing, and problem solving via a tactics game (see the Supplementary Information for the details of each module). All but the sorting game had three levels of difficulty.

#### Visual Search

Visual search involves searching among a matrix of cards for a feature or a combination of two features. Given the competition among members to win points, this exercise also engages processing speed. *Goal-setting arithmetic* required the participants to perform arithmetic computation to compete for being the first person who reached one of the pre-defined rules. To be the first person to reach a pre-defined goal, participants could engage in strategic thinking and problem solving.

#### Reasoning

Reasoning with hypothesis testing involved inferring a rule based on the feedback provided by a dealer who showed either the cards matching on the target feature or the cards mismatching on the feature. Thus, it required feature-attention, hypothesis-testing, and decision-making related to uncertainty. The *Working memory* game is similar to the delayed-matching-to-sample task by requiring the participants to find pairs that match on a feature in a matrix of cards. Thus, participants must pay attention to the features and maintain information in working memory. They could also adopt strategic thinking to hinder the chances for other participants to get pairs.

#### Updating

Updating was the n-back task, and the participants had to play a card that matched on the pre-defined feature the card shown by the previous *n*th player. Both a 1-back and a 2-back game were played. This task demands attention to features, maintenance of information in working memory, and updating information in working memory. *Tactics* activities modified the game UNO using the deck of person cards we designed. Participants had frequent opportunities to adopt strategic thinking based on the cards in hand. In this game, the participants were required to concentrate on features, maintain information in working memory, think strategically, and make decisions related to uncertainty.

#### Interfacing with Long-Term Memory

Interfacing with long-term memory was trained with sorting activities in which the participants were asked to sort a deck of cards with objects based on different categories (e.g., living or non-living, edible or not). In the dual-task sessions, a target (a city name or a name of an animal) was randomly selected by the trainer, who called out the target from time to time while the participants concentrated on the primary task. Participants were asked to raise their hands whenever the target name was called out. Thus, the secondary task requires maintaining a target in working memory and monitoring the presence of the target.

To incorporate elements that promote gaming motivation, all the games began with easy difficulty for the participants to easily master. In many games, participants took turns being the dealer in a run and could decide the strategies and goals on the fly according to the cards in hand. They were also teamed up while free discussion was encouraged in some games to satisfy the needs of relatedness. Positive and informative feedback was provided by revealing the accuracy of participants’ selections and decisions, with points earned as the bonus rewards.

### Board Games for the Active Control Group

Eight commercial board games were selected for training eye-hand coordination, rule maintenance, strategic thinking, reasoning and planning in the active control condition (see Supplementary Information for detailed information of each game). None of the participants had played these games prior to participation. As described in the Supplementary Information, all the games required participants to follow a task rule maintained in working memory. The goal in six games was to sustain the balance of an object (e.g., tree trunk, an inclined pizza) so that the participants must reason and decide which pieces to put or draw without breaking the balance. In the other two games, strategic thinking and decision-making were engaged in one context that occurred occasionally. No games involved executive control in updating, inhibition, or dividing attention between two task rules. Processing speed was not trained as the participants could take their time to choose their actions. The degree of training of executive control was less than the cognitive training condition.

### Outcome Measures

We included three categories of untrained tests to measure the training effects: executive control, reasoning, and delayed episodic memory. The first category of three tests shared overlapping cognitive processes with those underpinning the training activities. According to Zokaei et al. (2017), transfer should be observed when the transfer tests rely on neural mechanisms that underpin the trained activities. Given that we designed the cognitive training program to target executive control, we expected to find benefits in the first category of tests if the cognitive training program was effective.

**FIGURE 5:**
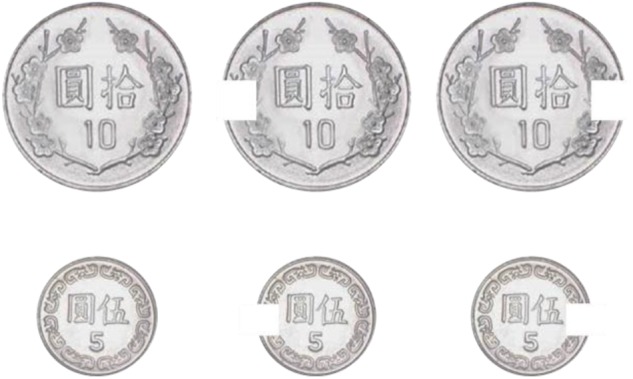
An illustration of the target and distractors used in the selective attention test.

Within the category of executive control, *selective attention* was measured with a counting test in which participants had to count, as quickly and as accurately as possible, the number of intact coins among coins with a gap in one side (Figure 5 for an illustration of the target and distractors). Because the distractors are highly similar to the target, this test requires the participants to engage in object-based attentional control, ignoring or inhibiting highly similar distractors, and maintain a running count in their working memory. The test is in paper-pencil form, with 18 targets among 32 similar distractors. Reaction time (RT) was measured by a timer.

#### Control of Content in Working Memory

Control of content in working memory was assessed by the backward digit-span test in the WAIS-III intelligence test battery. Participants were required to remember a series of digits and recall them in backward order. This test requires the participants to transform the digit sequence (Reynolds, 1997), which may engage visual strategies (Cornoldi and Mammarella, 2008). This test also requires shifting attention, inhibition, goal planning and monitoring (Bull et al., 2008).

#### Executive Function-Switching

Executive function-switching was evaluated by a modified color trail-making test (CTMT) in paper-pencil form. Participants were handed a piece of paper on which digits from 1 to 12 were embedded in black and white circles. Participants were asked to connect the digits in a sequential order while switching between the black and white circles. Multiple processes are involved in trail making, ranging from attentional shifting, scanning and searching, and attentional control (Lezak et al., 2004).

#### Reasoning

Reasoning was tested by the Raven’s Progressive Matrices (RPM, Raven et al., 1998) that require abstract reasoning, visuospatial ability and test-taking strategies (Hayes et al., 2015).

#### Delayed Episodic Memory

Delayed episodic memory was assessed with the accuracy in recognizing the contextual information in a story after a delay of 5 min. The story described a robbery event, contained 15 units of information with 4 units about people, 2 units about time, 3 units about places, 2 units about quantity, 1 unit about object, and 3 units about action. Two versions, with an equivalent event structure and number of units in the story, were tested for recognition at pretest and posttest.

### Procedure

One week prior to the training sessions, the outcome measures and the MMSE were assessed individually in a quiet corner of the community center during a period of few activities in the center. After a brief period of rapport-building, the participants first read and signed the informed consent form. The assessor then began the procedure of checking the fulfillment of the inclusion criteria, including the IADL and MMSE tests. Afterward, the assessor conducted the tests for measuring baseline performance. The assessor first read a short story and asked the participants to remember it for a later test. The assessor then conducted the selective attention test and the CTMT test. On each test, the participants first practiced on a short form before taking the test. The paper was facing down and flipped when the participants were ready. RT of the selective attention test was recorded by a timer which was activated when the assessor flipper the paper. The CTMT test had a time limit of 3 min. Upon completion, the participants could rest until 5 min had elapsed from the end of story reading. The assessor then conducted the RPM test. The pretest session took approximately 1.5–2 h. The outcome measures were again administered to participants in the training and active-control groups 1 week after the program. The same set of measures was administered to the waitlist control group with the same interval between the pretest and posttest.

#### Cognitive Training Program

The program was carried out in each community center. Five to six persons were assigned to one group and group membership remained the same throughout the training period. Each group was trained at different times of the week to reduce any intergroup interference, with 1 day intervening between the two sessions for distributed practice of cognitive functions. In each session, we first ran warmup activities for an average of 15 min (10–20 min in different sessions). Warmup activities were designed to tap the abilities of attentional focusing, executive functions, short-term memory of associations, and memory of related or unrelated items over a long period of time (See Supplementary Information for the details of each activity).

The order of arranging each module was fixed across the training groups for the reason that the participants could become familiar with features on the cards during early sessions and played games that involve more functions in later sessions. To familiarize the participants with the cards we designed, we adopted the visual search game in the 1st week. Except for tactics and sorting, each module was run in both sessions of the same week. The training of interfacing with long-term memory was run in only one session, because some participants found the activities less appealing and they were not motivated to continue the training for another session. Since tactics was favored by most of the participants, it was run for three sessions. Dual-task training was conducted in the last week, with the goal-setting arithmetic activities in one session and the tactics in another session as the primary game. Each training session lasted for 75 min, with a break of 15 min after 30 min of activities. Each session involved many runs of activities, the person who reached the goal first in a run earned a point, and the winner who accumulated the most points at the end of a session received a small gift (e.g., soap, a bottle of dishwashing liquid).

#### Active Control Group

All aspects were similar to those experienced by the training group except for the games played in the sessions. In the warmup period, the participants chat about recent events in their lives and which parts of the previous training activities were more appealing to them. As in the cognitive training condition, the winner who accumulated the most points in each session received a small gift.

#### Waitlist Control Group

No contacts were made between the pretest and posttest. Participants simply carried out their routines as usual. They served as volunteers in the center at least once a week.

## Results

The proportion of correct responses in accuracy was first calculated for each measure. We normalized the scores of each measure across the three groups for the pretest and posttest using the mean and standard deviation of 138 observations. Four measures related to executive control (RT of the selective attention test, accuracy of the selection test, backward digit span, and CTMT) were averaged as a composite score^3^. Analyses were conducted on the normalized scores using a 3 (group: training, active-control, waitlist-control) x 2 (test time: pretest, posttest) mixed-design analysis of variance (ANOVA).

### Executive Control

**FIGURE 6:**
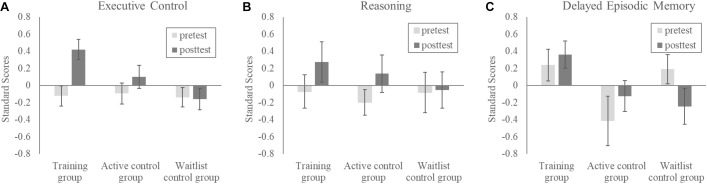
The standardized scores as a function of group and test time on **(A)** the composite measure of executive control, **(B)** the measure of reasoning, and **(C)** delayed episodic memory.

The results showed a significant interaction between group and test time, *F*(2,66) = 7.84, *p* < .001, $\eta_{p}^{2}$ = 0.19. The simple main effect of test time showed that the cognitive training group significantly improved on executive control, *F*(1,66) = 28.57, *p* < 0.001, $\eta_{p}^{2}$ = 0.30, the active control group showed a trend of improvement, *F*(1,66) = 3.61, *p* = 0.06, $\eta_{p}^{2}$ = 0.05, and the waitlist group did not show any improvement (*p* = 0.84). Figure 6 shows the normalized scores across three groups at the pretest and the posttest. The results of following two-way mixed-design ANOVAs showed that the Group x Test time interaction was not significant when comparing the two control groups (*p* = 0.18), significant when comparing the cognitive training group with the active control group, *F*(1,44) = 7.40, *p* < 0.01, $\eta_{p}^{2}$ = 0.14, and when contrasting the cognitive training group with the waitlist control group, *F*(1,44) = 14.61, *p* < 0.001, $\eta_{p}^{2}$ = 0.25.

### Reasoning

The results (Figure 6) showed a significant main effect of test time, *F*(1,66) = 6.29, *p* = 0.01, $\eta_{p}^{2}$ = 0.08 and the other effects were not significant (*p*s > 0.30). All participants improved from the pretest to the posttest.

### Delayed Episodic Memory

The analysis did not show any significant effects (*p*s > 0.05). The cognitive training group did not outperform than the other groups.

## Discussion

The aim of this study was to investigate whether a group-based multi-component cognitive training with social engagement can improve executive control in older adults. We developed warm-up activities and card games based on theoretical models of executive functions. Participants went through 8 weeks of cognitive training, with two sessions a week and 1.5 h per session. To examine whether social engagement with activities targeting cognitive functions at a low-demand level, we also included an active control group. A group of volunteers served as a waitlist control condition to investigate test-retest effects. The results showed that only the cognitive training group improved significantly on executive control. The active control group showed a trend for improvement, and the waitlist control group did not show any changes in the functions related to executive control. When contrasting the cognitive training group with each of the two control groups, the effect size was larger compared with the waitlist control group. All three groups showed significant improvement in reasoning and not on the test of delayed episodic memory.

## Social Engagement on Cognitive Functions

The results echo the suggestion that an active (or a treated) control group is necessary to evaluate the effects of a training program (see Melby-Lervåg et al., 2016 for a review), as the effect size was larger when contrasting the cognitive training group with the waitlist control group. Nevertheless, the inclusion of a waitlist control group still has its merits as it allows the examination of cognitive performance after a period of daily activities without any specific cognitive intervention and rules out the possibility that the improvement results from being familiar with the test. All three groups showed improvement on the test of reasoning, suggesting a role for familiarity.

Note that the participants in the waitlist control group volunteered at least once a week, and they went to the community center frequently. Correlational studies have shown that volunteer work protects against cognitive aging (see Anderson et al., 2014; Guiney and Machado, 2017; Proulx et al., 2017 for reviews). The results of the Experience Corps project (Carlson et al., 2008, 2009) have shown the benefits of volunteer work for those who showed impairment at baseline before joining the project. Volunteer work also led to a medium-sized but non-significant neural effect at a 2-year follow-up (Carlson et al., 2015).

Two causes may explain the finding of no improvement on executive control in the waitlist control group. First, the volunteers recruited were active and were not impaired generally in cognitive functions. Second, the cognitive processes required in their volunteer work may not have imposed adequate challenges to the neural systems to trigger any change in the plasticity or efficiency of neural processes related executive control. The participants helped visitors in various activities (e.g., paperwork for using facilities) and served meals for other elders coming to the center. They also participated in various social activities (e.g., field trips) with others as a group. Social interaction characterizes their volunteer work. By contrast, the participants in the Experience Corps project were trained to assist with a variety of activities, such as reading and literacy skills practice, library support, violence prevention, community outreach, and behavior management. They also committed 15 h per week of service for at least 1 year (Glass et al., 2004). The finding of intervention effects on the hippocampal volume (Carlson et al., 2015) may have arisen from the remembering and execution of many new skills. The activities and cognitive processes involved in the volunteer work should be clearly charted in future research to investigate its effects on cognitive functions. Productive engagement rather than receptive engagement is more beneficial for cognitive functions (Park et al., 2014). Productive engagement at a less-demanding level can lead to some improvement on the trained cognitive functions, as the active control group showed a trend for improvement on executive control. The effect arose because participants were required to maintain the task rule in working memory and reason through action plans for all games. Additionally, they were engaged in hypothesis testing in certain contexts.

## Near, But Not Far Transfer

Although we adopted untrained tests for measuring the training effects, the benefits should be taken as a near transfer when the trained functions and the outcome measures share overlapping cognitive processes, and therefore, engage overlapping neural mechanisms. Zokaei et al. (2017) argue that far transfers without overlapping neural systems may not make sense from a theoretical viewpoint. Supporting this view, we observed a significant transfer effect on executive control in the training group and a marginal effect in the active control group. The improvement may have resulted from repeated challenges to the cortical circuits sub-serving trained processes as expected by the cognitive reserve hypothesis (Whalley et al., 2004) or from increasing the plasticity of the cortical circuits in response to training activities as hypothesized by the STAC-r model (Reuter-Lorenz and Park, 2014). By contrast, null group effects were observed in the test of reasoning and the test of delayed episodic memory. The null effects arise because the cognitive processes underlying these two tests were untrained.

We incorporated a module on practicing reasoning and a module of tactics that requires strategic thinking. The trained activities may not fully engage the cognitive processes that are critical to problem solving in the RPM test. The trained activities require reasoning based on feature attributes without relational integration. By contrast, the RPM test requires relational integration at abstraction to solve each problem. Similarly, the null group effect on the test of delayed episodic memory may have arisen from not engaging the critical cognitive processes required in performing the test. Although the participants engaged with activities requiring memory processes in most of the warmup sessions and with a module of short-term memory in formal training, the contents they were required to remember do not contain many contextual elements. Another reason for not observing benefits on the delayed episodic memory is that we never trained the participants on using different mnemonics for encoding contextual information. A third reason is that the story is too short with only 15 units of information for measuring the changes after 5 min of distraction. Very few participants in each group scored less than 80% of accuracy at the pretest. The ceiling effect may underlie the null results.

Integrating results across studies of the Experience Corp project (Carlson et al., 2009), new learning (Chan et al., 2014; Park et al., 2014), social engagement (Stine-Morrow et al., 2014), and the results of the current study suggest that the cognitive processes involved in social engagement determine whether an intervention could be beneficial to cognitive functions. Benefits for cognitive functions occur only when the cognitive processes trained in social engagement trigger plasticity or efficiency in the underpinning neural systems. Therefore, transfer occurs only when the outcome measures engage cognitive processes that rely on the neural systems activated by the trained activities (Zokaei et al., 2017).

The NeuroRacer game, for example, trains older adults to coordinate a tracking task and a signal detection task (Anguera et al., 2013). The far transfer to tests of sustained attention and working memory may have arisen from cognitive control processes that activate neural mechanisms that are similar to the ones engaged during training. The dual-task training requires the maintenance of task rules in working memory (e.g., only respond when the green circle occurs, keeping the car on the cross cursor), focused attention on matching the car onto the cursor, inhibition of responses when non-targets occur, and switching attention between the tracking and detection tasks.

Similarly, productive engagement in new learning (Park et al., 2014) and in solving ill-defined problems (Stine-Morrow et al., 2014) enhances cognitive functions that share overlapping processes with the trained activities. For example, learning digital photography requires remembering contextual information (e.g., when to use a function) and sequential organization (e.g., the steps of achieving a goal). As a result, participants who learned digital photography improved more on episodic memory (Park et al., 2014). Participants who worked on solving ill-defined problems improved on divergent thinking (Stine-Morrow et al., 2014). The mechanisms underlying the improvements on executive functions after learning to use Facebook (Myhre et al., 2017) remain less clear.

## Multi-Component Cognitive Training with Social Engagement

A group-based cognitive training has many drawbacks. First, it is difficult to match the intelligence or cognitive functions among group members. Second, it is difficult to control the trained content with precision, because group dynamics can easily alter the progress. The sorting game was run in only one session because some participants expressed reluctance to play the game, and their attitudes influenced other group members. Third, each training session may need to blend different training activities to sustain motivation. Fourth, it requires a trainer who is good at interacting with older adults and who can adjust the training activities on the fly. Fifth, an interactive, group-based training cannot adapt task difficulty for each participant. The training cannot be personalized, which is considered important for improving cognitive functions (see Bamidis et al., 2015; Zokaei et al., 2017). Lastly, it is more difficult to keep the same members in each group for scheduling, and a long period of training is unlikely to recruit participants.

Despite the many disadvantages of group-based, multi-component cognitive training, the current study shows promising results for promoting group-based cognitive training in community settings. Note that we designed the games based on theoretical models of executive control rather than using traditional tasks used in laboratory settings that are more appropriate in a one-to-one context. The results are in line with the STAC-r model (Reuter-Lorenz and Park, 2014) and the cognitive reserve model (Whalley et al., 2004) in which the plasticity and efficiency of cortical circuits play a key role in maintaining cognitive functions in older adults. An intervention may be effective only when the trained cognitive processes impose sufficient challenges to the underlying neural systems. Without the activated plasticity or efficiency in the neural systems, older adults may simply adopt their usual processing strategy to meet the demands in everyday life. The “use or lose it” philosophy stands valid. Using card games to teach and practice strategies for attention, control, memory, and solving complex problems with adaptive training can be beneficial to older adults in community settings where people gather and interact.

It should be noted that cognitive training does not always bring near transfer effects especially when the training effect is tested years after the intervention. Even with boosting sessions before assessment, training of reasoning and of memory functions does not lead to any near transfer effects 10 years after the intervention (Rebok et al., 2014). Meta-analysis studies also showed small effect sizes of near transfer (Karr et al., 2014; Kelly et al., 2014). An effective approach could be integrating theoretically based multi-component interventions that engage the neural mechanisms involved in daily activities. Moreover, many factors could influence the outcome of an intervention program, including participant variables (e.g., education, personality traits related to motivation and need for cognition, life style), intervention variables (e.g., the duration of the intervention over time, the dosages of the intervention each time, spaced versus massed intervention). The factors of these two categories could interact to influence the outcome of an intervention program. Future research should systematically investigate how these variables interactively modulate the outcomes of a cognitive training program and the neural mechanisms that support the outcomes.

## Limitations

Seven limitations are acknowledged. Firstly, the sample size of each group is small. Although we reached the minimum requirement of 20 subjects per condition (Simmons et al., 2011), the sample size is rather small compared with studies of new learning or social engagement using a large sample (Park et al., 2014; Stine-Morrow et al., 2014). The relatively small sample size could limit the ability to observe significant effects when the effects are small. Second, we did not include a group with pure cognitive training for comparison. It is uncertain whether the benefits observed in the cognitive training group could exceed cognitive training alone. We did not include this group for a few reasons. We were motivated to promote cognitive training in community settings. Many games could not be played alone, and it is more fun to play with others. Additionally, social interaction is inevitable, even in a one-to-one customized training program.

The third limitation in the current study is the usage of a subgroup of tests for measuring executive control but only one test for measuring reasoning and one test for assessing delayed episodic memory. Additionally, we did not include measures of cognitive performance in real-world scenarios. We did so to minimize fatigue and impatience while focusing on the measures that share overlapping cognitive processes with those underpinning the trained activities. The fourth limitation is the null evidence that the degree of social engagement was comparable between the training and the active control groups. Thus, differences in motivation may also underlie the results. The fifth limitation is the inability to conduct a follow-up study to investigate whether the training effects can be maintained over time. We could not fulfill this plan because not all participants could commit to a follow-up study. The sixth limitation is that processing speed was trained in the cognitive training group but not in the control groups such that the improvement in processing efficiency could contribute to the benefits in executive control. Lastly, we did not conduct neuroimaging studies to validate our manipulations to confirm the change in neural efficiency or plasticity. Therefore, we could not verify whether compensatory processes indeed occur for testing the scaffolding framework and the cognitive reserve model. Without this information, it is unknown why the training benefits executive control in behavioral measures. Future research should investigate whether multi-component cognitive training with social engagement indeed triggers changes in efficiency or plasticity in the underlying neural systems to produce training benefits.

## Conclusion

We tested whether a group-based multi-component cognitive training can lead to benefits for older adults. We designed a battery of card games and warmup activities based on theoretical frameworks of executive control. Given the shared cognitive processes, this cognitive training leads to benefits for executive control via repeated challenges to these processes. However, we did not find transfer effects for reasoning or delayed episodic memory as these two functions were less trained. Future research may incorporate games and activities that trigger efficiency and plasticity in the neural systems supporting these two functions. For adaptive training to be more effective in a group-based intervention, participants may need to be matched on core functions, such as working memory capacity. Systematic research in this area may provide valuable guidelines for taking the interventions to community settings as a choice for older adults to maintain or improve their cognitive functions in various domains.

## Author Contributions

Y-YY, C-YK, and Y-MH developed the study concept and design. Data was collected and analyzed by C-YK. All authors interpreted the results. C-YK drafted the manuscript under the supervision of Y-YY and Y-MH, and Y-YY provided critical revisions. All authors approved the final version of the manuscript for submission.

## Conflict of Interest Statement

The authors declare that the research was conducted in the absence of any commercial or financial relationships that could be construed as a potential conflict of interest.

## Footnotes

1 We distinguish multi-component cognitive training from new learning as two different routes of intervention. The former adopts a theory-driven approach with specific functions (e.g., inhibition, switching) in mind. The latter involves learning a new skill (e.g., digit photograph) that may engage many different functions. A task analysis is required to uncover which cognitive functions are trained in new learning, and whether the task analysis is valid requires verification from the training outcomes.

2 We asked four experts in cognitive science to provide independent ratings of each game module. Their feedbacks validate the engagement of multiple cognitive functions in each game played in the training group and the less involvement of executive control in the games played in the active control group.

3 As RT has higher resolution and is more sensitive to changes compared to accuracy, we excluded RT from the composite score in another analysis. The results remained the same.
