# Supplementary material for: Let’s Play Cards: Multi-Component Cognitive Training With Social Engagement Enhances Executive Control in Older Adults
Source: Front Psychol. 2018 Dec 6;9:2482. doi: 10.3389/fpsyg.2018.02482 (PMC6291491; doi:10.3389/fpsyg.2018.02482)
Supplement: Supplementary file 1 [file Table_1.pdf]

Supplementary Information

Formal training

The following table summarizes the cognitive functions engaged in each training game and the number of sessions each game was played. The details of each game are then described. All games involve maintaining and following rules, attention to features, and decision making.

Game	Session	Additional cognitive functions
Visual search	2	processing speed, color matching, working memory, strategic thinking, monitoring of target presence, hand-eye coordination
Goal-setting arithmetic	2	working memory, mental calculation, updating, strategic thinking in problem solving
Reasoning	2	working memory, updating, hypothesis testing, decision-making related to uncertainty, monitoring of target presence
Working memory	2	working memory, color matching, divide attention between two task sets, strategic thinking, monitoring of target presence
Update	2	working memory, color matching, updating
Tactics	3	working memory, strategic thinking, decision-making related to uncertainty, monitoring of target presence, switching to secondary task, dividing attention between two task sets
Sorting	1	interfacing with long-term memory, strategic thinking in problem solving
Dual-task	2	maintain and divide attention between two task sets in working memory, monitoring of target presence, switching to the

secondary task when required

Visual search. At the beginner level, 32 cards were face-up on the table. Each participant drew one feature card (e.g., a red T-shirt) and put it on the table. Participants took turns in searching for the cards containing their target feature. At the intermediate level, each participant drew two feature cards with one containing a feature and another without a feature (e.g., red T-shirt and no glasses). At the third level, the number of faced-up cards increased to 64. Once participants became familiar with the rules, they competed to collect as many cards as possible. They could strategize by drawing a card with a feature that matched another participant's target feature so that the other person would have one less card to draw.

Goal-setting arithmetic. At the beginning level, the trainer dealt four cards to each participant and put the rest face down. Beginning from 15 or 35, each participant took turns playing a card and was required to saying aloud the outcome of adding or subtracting 5 depending on if the T-shirt color was red or blue. Then, each participant drew a new card. The goal was to be the first person to reach 0, 25, or 50; and the winner would take all the cards on the table. The person who got most of the cards at the end won a run. The strategy was to prevent the next participant from reaching the goal. At the intermediate level, the initial number changed to 25. Each person could choose to add or subtract the digits on a card to get 25, less than 0, or greater than 50. At the third level, the participants were paired in teams. Moving clockwise or counter-clockwise, the first person in the team should help the second person without knowing what cards he/she had, and the second person should adopt a strategy to prevent the next team from winning. In the dual-task session, a target (a city or a fruit name) was pre-defined, and the participants were asked to clap their hands whenever they heard the target while playing the cards at the third level.

Reasoning. The trainer first handed five Lego pieces as tokens to each participant. Participants took turns being the dealer for each run. The dealer would have one target feature in mind and play one card at a time, up to five cards in total, and other participants would guess the target feature. Before guessing, a person would need to pay one token. The dealer provided feedback following each guess, and the person who guessed correctly collected all the tokens on the table. At the beginner level, the dealer would put the card with the feature in one pile and the card without the feature in another pile. At the intermediate level, the dealer only revealed the cards containing the feature while participants were paired and could discuss their guesses. At the third level, the dealer only revealed the cards without the feature.

Working memory. The trainer put 16 cards face down on the table. Each participant drew a feature card and then flipped over two cards at a time until he/she found two cards that matched the feature card. The cards would be placed face down if one card did not match on the target feature. If a person decided that there were no more cards on the table that contained the target feature, he/she would need to put two Lego tokens on the table and raise his/her hand for an announcement. If this person was correct, he/she would win two extra tokens, and if incorrect he/she would lose the two tokens. The person who got the most matches was the winner. At the intermediate level, the number of face-down cards increased to 32, and each person drew one card containing a feature and one card without a feature for the search goal. At the third level, the number of face-down cards increased to 64.

Updating. Each person was handed 8 cards and the rest of the cards were face down in a draw pile on the table. The first person turned over the first card and revealed it to all participants and then put it face down in the discard pile. The participants took turns playing cards. The second person then played a card that matched the first card on one feature, named the feature for accuracy, and put it face down on the discard pile. The next person then played

a card that matched one feature of the second person's card and put it face down in the discard pile. If a person did not have a card that matched on any of the six features, he/she drew a card from the draw pile. The first person who played all the cards in his/her hands won a run. At the intermediate level, each person was handed 4 cards and the rule changed from 1-back to 2-back so that the first person played two cards from the draw pile at the beginning. At the third level, matching was made on two features under the 2-back rule. Participants could interfere with others by saying a feature that did not match the one the person was trying to name.

Tactics. Every participant started with five cards, and the rest of the cards were placed in a draw pile face down. The first person dealt the top card and placed it face up in the discard pile. The next player played one card that matched on one feature and put it on the top of the discard pile. A person would need to draw one card if he/she did not have a card matching on that feature. The target feature remained the same until a person played a card that matched the top card on three features. This person could change the rule as he/she wished. A player was required to announce when only one card was left in his/her hands; if someone played the last card in his/her hands without announcing beforehand, the player lost the chance to be the winner. The first person who played all the cards in his/her hands won a run. The four jokers could be played anytime, and players could also change the target feature with a joker. At the intermediate level, a card with a number that was a multiple of 5 (e.g., 5, 15, 25) could be played as a *Framed* card, which allowed a person to prohibit the next person from playing a card and to draw two cards from the pile. If the next person also had a *Framed* card, the one after him/her had to draw four cards from the pile. Additionally, a card with a units-digit 3 could be used to reverse the direction of playing. At the third level, a person who played a card with a units-digit 7 could exchange all the cards in his/her hands with any other player in

the group. In the dual-task session, the participants were required to clap their hands whenever they heard a target city or fruit while playing the game at the third level.

Interfacing with long-term memory. The participants were asked to classify the cards into two categories at the beginning. The cards were then re-categorized into three piles by another rule and a run continued until the participants could not come up with anymore new rules. Participants could discuss how to sort the cards to encourage brain-storming.

Warmup activities

Although the activities also engaged attentional, control, and memory processes, we did not consider them part of the training intervention, because the duration was quite short and less structured. The total duration was 30 min for two sessions on switching and 50 min across three sessions on inhibition. The other activities were related to the maintenance of various forms of information. The trainers, as they prefer, could choose which form of information to be maintained in each session.

Binding and disjunction. This activity modified the commercial Geistesbliz game. The trainer first laid five target cards on the table (grey elephant, blue cloud, green vegetable, yellow T-shirt, and red umbrella). The trainer then showed one card with two objects in two different colors. If one object was in the right color (e.g., green vegetable), the participants were required to pick up the target card from the table. If both objects were colored incorrectly, the participants needed to find the target card that did not contain any of the colors or objects. For example, a correct answer would be a yellow T-shirt when the trainer showed a card with a blue vegetable and a red elephant. At the first level, the participants were required to name the target on the card while they selected it. At the second level, they were asked to say “white elephant” regardless of the correct target object. The participant who first picked the correct target card was the winner in that round.

Short-term/working memory. At the beginning level, the trainer first informed the target in the subsequent series of cities or fruits. The trainer then named the cities or fruits; the participants were required to clap their hands whenever the target was named. At the intermediate level, one city and one fruit served as targets. At the third level, the number of city and fruit increased from three (two cities and one fruit; one city and two fruits) to four units (two cities and two fruits).

Rule maintenance. Participants took turns calling out numbers in order beginning with 1. At the beginning level, the participant would clap his/her hands when the number could be divided by three (e.g., 3, 6, 9…) or contained the units-digit 3 (e.g., 3, 13, 23, 33…). At the intermediate level, the rule changed to a number that can be divided by three or four (e.g., 3, 4, 6, 8…); contained the units-digit 3 or 4 (e.g., 3, 4, 13, 14…). At the third level, the rule remained the same, but the participant should kneel while calling out the number.

Maintenance of action sequence. The trainer first demonstrated a sequence of actions, and the participants were asked to imitate the sequence afterwards. The sequence length increased over time. At the second level, they were asked to reverse the sequence in imitation; the sequence length also increased over time.

Maintenance of number – action pairs. The participants were required to remember four number-action pairs (e.g., 1: raise right hand). The trainer then spoke out a number randomly selected from 1 to 4; the participants were required to perform the corresponding action. At the second level, the trainer would name a series of digits randomly mixing the four digits for the participants to execute the corresponding actions. The length of a series increased over time. The action could change in latter runs.

Executive function – switching. Two triangular cards could be connected on one side by matching on the color, shape, or digits. Participants were encouraged to switch dimensions as

much as possible and connect as many cards as possible. They began the game individually and then worked together to connect as many cards as possible.

Executive function – inhibition. This exercise modified the *Simon says* game played by children. The trainer took the role of “Simon” and issued an instruction that the participants must either follow (e.g., raise the right hand) or inhibit the responses. At the second level, they also needed to remember that “red hand” referred to the left hand and “yellow hand” referred to the right hand.

Delayed memory. Various activities were run in different sessions. In one type of session, the trainer would name the items to be remembered (TBR) before training began, and the participants were asked to recall the items at the end of training. The items consisted of unrelated objects or idioms. In the second type, TBR could be the story of a target person’s experience one day, the tax identification number in 8 digits for each of three schools, or the three meals a person had in a day. The participants were asked to retrieve the answer given the question the trainer asked (e.g., what did he have for lunch). In the third type, three objects or three Chinese Zodiac signs were the TBR; the participants were asked to recall the three items according to their relative sizes or ranks in the Zodiac system. In the fourth type, a series of 8 digits was the TBR. Easy tasks were run during the early weeks of the training program and the more difficult tasks were run during the later weeks.

Commercial board games

The following table summarizes the cognitive functions engaged in each commercial board game and the number of sessions each game was played in the active control group. The details of each game are then described. The name of each game is replaced with one that describes the essence of the game. All games involve maintaining and following rules. All except for the modified Monopoly game engage hand-eye coordination, decision making, and

fine motor skills.

Game	Session	Additional cognitive functions
Tree growing	2	none
Bark chopping	2	none
Honey taking	1	color matching, strategic thinking
Log knocking	1	strategic thinking
Tower building	3	color matching, strategic thinking
Pizza making	2	strategic thinking
Match & Squeeze	2	color matching, strategic thinking
Monopoly	3	mental calculation

Tree growing. A tree trunk with holes was set first. Each participant was handed pieces of branches and leaves. The participants took turns putting the pieces into the trunk. The goal was to put all the pieces into the trunk while maintaining its balance. The one who broke the balance lost the game; the person who put all the pieces into the trunk won the game.

Bark chopping. A tree trunk with removable barks was set first. Participants took turns in chopping the bark with an axe twice each time until all the bark was chopped. The goal was

to maintain the centerpieces of the trunk while chopping. The one who chopped most of the bark won the game.

Honey taking. A honeycomb of colored hexagonal cells with a bee sitting on the top was set first. Participants took turns in spinning a spinner to determine which colored cell they were going to knock out of the honeycomb, which destabilized the inner structure of the honeycomb. The goal was to keep the bee on top while knocking off the cells. The first person that let the bee fall lost the game.

Log knocking. A beaver sitting on top of log pieces was set first. Participants took turns pushing a piece out of the log pile while keeping the beaver on the top. The first person that moved the beaver down to the bottom successfully won the game.

Tower building. A tower with 18 layers of three wooden blocks was set first. Participants took turns removing a single block from the tower anywhere below the highest layer with three blocks and then put the block on top to form a new layer on the top of the tower. The goal was to continue building the tower while keeping the tower balanced and intact. The one who made the tower fall lost the game.

Pizza making. A pizza man holding an inclined pizza with a finger was set first. Participants took turns putting all the toppings (tomatoes, mushrooms, peppers and pepperoni) on the pizza while keeping the pizza balanced. The one who let the pizza fall lost the game.

Match and Squeeze. Each participant was handed a log, a squeezer, and five colored acorns. Participants took turns in spinning a spinner and taking the action shown on the spinner. When spinning ended on a particular colored stripe, participants need to squeeze the matching colored acorn with the squeezer and place it into the log. When the spinning ended with a symbol showing a squirrel thief, they could exchange their log with any player. They

took acorns out of the log when the symbol showed wind blowing. The first person that filled the log with all acorns won the game.

Monopoly. All, but one, aspects were the same as played in the typical game with the goal of bankrupting opponents. The decisions to buy, sell, or trade a property were based on the parity of the number rolled on the two dice rather than on one's own decision. Participants chose one of the properties to sell when there was no ready money. The one who went bankrupt lost the game, and the one who had most of the property was the winner.
